# Development and Performance Evaluation of Biomass-Based Injera Baking Gasifier Stove: A Case Study of Clean Cooking Technologies in Ethiopia

**DOI:** 10.1155/2024/1524398

**Published:** 2024-04-08

**Authors:** Dawit Tessema Ebissa, Eshetu Getahun

**Affiliations:** ^1^Bahir Dar Energy Center, Bahir Dar Institute of Technology, Bahir Dar University, Bahir Dar, Ethiopia; ^2^Ethiopian Textile and Fashion Design Institute, Bahir Dar University, Bahir Dar, Ethiopia; ^3^Chemical Engineering Department, Bahir Dar Institute of Technology, Bahir Dar University, Bahir Dar, Ethiopia

## Abstract

The primary energy source in Ethiopia is biomass. Over 80% of Ethiopians are rural dwellers who rely on biomass energy for lighting and cooking. In most parts of Ethiopia, injera is traditionally baked using an open fire, a three stone, or a device using woody biomass. These baking stoves have very low efficiency and consume a significant amount of fuel. Moreover, these traditional baking stoves have released large amounts of indoor air pollution, which has led to different types of health-related risks, especially for women and children in the country. Therefore, the aim of this study was to investigate efficient and fuel-saving injera baking technologies. Rigorously, an injera baking gasifier stove was designed, developed, and characterized in detail through water boiling and control cooking test methods. The indoor air pollution level was evaluated using particulate matter measuring instruments. The result indicated that the developed gasifier stove had a thermal efficiency of 21.8%. Furthermore, an 86% fuel savings performance was demonstrated by the controlled cooking test for the injera baking gasifier stove. The average emission concentrations of particulate matter and carbon monoxide were 608 *µ*g/m^3^ and 9 ppm, respectively, during indoor air pollution determination. The study showed that injera baking gasifier stoves are a promising cooking technology for societies where baking is mostly dependent on traditional biomass fuel.

## 1. Introduction

With the anticipated population growth and the expansion of energy-dissipative financial activities in the long term, the demand for electricity globally is already rising and is expected to continue to do so [1]. Fossil fuels continue to govern most of the energy market, despite significant advancements in the renewable power era. They are inextricably tied to greenhouse gas (GHG) emissions and weather variations [1]. Approximately 1.2 billion people worldwide lack access to electricity, while 4 billion people cook with biomass [2]. The traditional and ineffective use of biomass leads to several significant problems, including the estimated four million fatalities per year that are linked to the poor health effects of indoor air pollution. Due to the use of biomass in their kitchens, particulate matter is present in almost 50% of early mortality of children under the age of five [2]. When cooking, it is common to use crude stoves or open fireplace pits with little ventilation and inefficient combustion, which exposes people to air pollutants both acutely and over time (particulate particles, carbon monoxide, nitrous oxides, carcinogens, and others) [3]. The main source of the adverse health effects is inhaling highly fine soot particles with an aerodynamic diameter of less than 2.5 *μ*m. In addition to having detrimental effects on fitness, traditional stoves frequently have negative social effects. For instance, inefficient stoves take longer time to bake injera and get gas, and the task is often carried out by women and children, taking time away from school and sports that generate revenue. Additionally, cook stoves powered by biomass and fossil gas provide 22% and 7% of the world's carbon emissions, respectively, making them the second most significant contributors to modern global warming [4]. Over the past four decades, various enhancements have been made to biomass gasifier-based cook stoves, aiming to simplify the cooking process for families and communities [4, 5].

Ethiopia's energy consumption is mostly dependent on biomass strength sources; 95% of this comes from primary power intake from biomass assets, with the remaining 4% coming mostly from contemporary strength sources such as electricity and petroleum fuels [6]. The national electrification charge in Ethiopia is at 25%, with 85% of the country's areas being urban and 10% being rural [2]. Ethiopia is consequently expected to depend on biomass as its primary source of strength for a considerable amount of time [3]. The main causes of Ethiopia's continuing deforestation and environmental degradation are the country's increasing demand for woody fuel and inefficient use of household biomass power, which results in significant power loss from cooking, baking, and lighting fixtures.

More than 90% of the final energy intake in Ethiopia is obtained through cooking, making it the primary energy-intensive technique. The baking of injera requires temperatures ranging from 180°C to 220°C [7, 8]. Over 98% of the family's consumption in the region is based on using traditional biomass for cooking; on average, a family uses 37% of their power usage to bake traditional staples known as injera [8].

The widely consumed and cultural meal of several East African countries, especially Ethiopia, Eritrea, and, to some extent, Somalia and Sudan, is injera, a processed food made of various cereals, such as teff, millet, sorghum, maize, wheat, and rice, or combinations of these enhanced through fermentation and rigorous baking techniques [9–11]. In Ethiopia and several other parts of East Africa, it is mostly consumed as a staple food.

**Figure 1:**
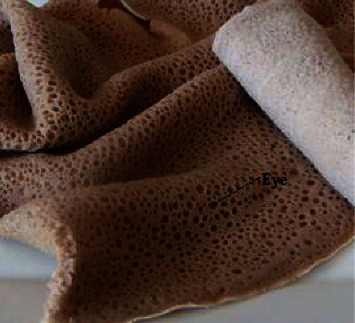
Texture of injera.

To make injera, we combine teff and other cereal flours with a sufficient amount of water and yeast. We allow the mixture to ferment for two to four days. To produce injera, the fermented dough was combined with hot water to maintain its optimal rheological properties.  Figure 1 illustrates how fermented doughs, when placed on a heated clay pan and kept at boiling temperature, release bubbles from the boiling water, creating mounds of small craters (eyes) at the top surface that give the unique injera texture an upward push. Pouring the dough directly into a heated clay pan requires careful consideration of the dough's thickness (thin layer) and fluidity. The blending ratio and fermentation time highly affect the textural properties and eye distribution of injera. If the thickness does not decrease, the cooked dough will turn into bread instead of injera. Injera is not eaten by itself like bread; instead, it is consumed with a few additions and stew, which we locally refer to as “Wat.” It is created with high-quality vegetables, grains, and meat products, and it is flavored with a spice combination known as berbery. Ethiopians consume injera, a staple food, in batches of 25 to 30 pieces at least twice a week, entirely depending on the diversity of individuals in each household.

Unprocessed petrol, wood, charcoal, animal dung, and other agricultural waste are the main traditional gas sources for injera baking. This is particularly common in developing countries like Ethiopia [12, 13].

**Figure 2:**
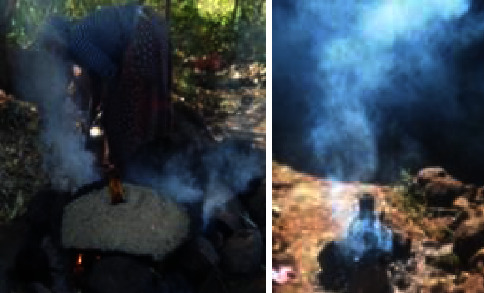
Traditional open fire (three-stone).

The majority of households complete their injera baking system by using an open fireplace, three stones, or a baking device, which is a wasteful and ineffective baking technique. The most in-depth household interest is the injera baking method, which accounts for about 50% of the biomass power consumption per family over a 12-month period [8, 13]. Furthermore, open fire pits and basic baking stoves expose people to air pollution (nitrous oxides, carbon monoxide, particulate matter, carcinogens, and others) both acutely and continuously [14, 15]. Consequently, family air pollution exposure has been connected to a number of detrimental health outcomes in both children and adults, including low birth weight, premature death, lung cancer, TB, pneumonia, and chronic obstructive pulmonary disease [16]. Conventional injera baking stoves frequently have negative social effects in addition to detrimental health effects. For example, inefficient baking stoves take a longer time to bake and produce gas, a baking burden typically given to women and children. Local forest deforestation and air pollution are the main environmental impacts associated with the traditional baking process [17].  Figure 2 illustrates how burning biomass using three-stone methods significantly releases air pollution in Ethiopia. This image was captured in rural Ethiopian households to highlight the effects of three stones on indoor air pollution levels and to show the suffering of women and children during the cooking process. Additionally, the unsustainable use of fuel wood damages nearby forests, contributing to the nation's high deforestation rate [18].

With an efficiency of only 5–10%, this traditional three-stone baking stove appears to operate at a relatively low level, leading to considerable fuel wooden consumption and thermal energy loss throughout the injera baking process [19, 20].

One of the major consumers of fossil fuels is the household sector, which struggles with issues such as access to stable, affordable, and smooth energy; poor stop-use efficiency of domestic appliances made locally and imported; and exposure to indoor pollution that poses health risks, especially to women and children. To address those issues, the units that use Ethiopia's national policy coverage on biogas include developing a variety of family friendly fuels and baking technologies, as well as advanced and energy-efficient lighting and household biogas systems. We promote era switching and localization [21]. Ethiopia's Climate Resilience and Green Economy (CRGE) Policy is the most recent legal instrument; it was introduced during the Organization's 17th meeting. By 2030, middle-class development is intended to be attained through a green economy and climate change. The National Clean Cook Stove Program of Ethiopia (NCCSPE), funded by the National Growth and Transformation Plan (GTP), recognizes that fuel woods serve as many people as a primary source of strength and respond to their needs for strength while also meeting the needs of country families. However, health concerns that govern air pollution in the home plague rural communities cook their food over open fires. The first-ever use of allure strength tactics was made possible by the Transitional Government of Ethiopia (TGE). Through the advancement of agroforestry, increasing the efficacy of biomass fuels, and encouraging the transition to better use of modern fuels, it seeks to solve concerns about household strength [22–24]. To lighten various challenges around cooking stoves, the Ethiopian government has launched a green saving plan meeting with others on the care of woodlands, expanding power result from energy from undetectable sources for domestic and local markets, and advancing energy-effective technology in transport, corporations, and buildings. The distinguishing habits in which the strength area is making use of developed management have a big impact—beneficial or negative and concerns like a shift of cooking technology out of inefficient and forest fuel-urgent stoves towards more efficient stoves and nonforest fire stoves vastly decrease the shame of woods [25].

Improved biomass cooking stoves were introduced to Ethiopia in the early 1980s in an effort to reduce indoor air pollution and deforestation [2]. Currently, the GIZ and international companies such as lakech, gonze, mirt, and rocket (tikikil) stoves are promoting and distributing cooking range innovations. However, because of incomplete combustion (direct combustion) of the biomass, these stoves are no longer just less environmentally friendly but also produce excessive amounts of indoor air pollutants.

**Figure 3:**
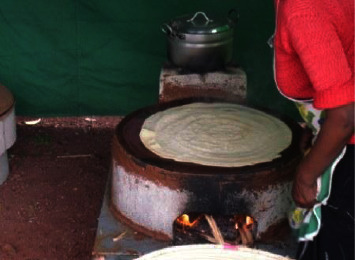
Improved injera baking mirt stove.

As shown in Figure 3, one of these enhanced cooking technologies is the use of a mirt injera baking burner. Mirt stoves have fuel-saving and thermal efficiencies of 40–50% and 16–21%, respectively [20].

Despite being an improvement over the three-stone stoves, the mirt stove's poor performance and high thermal energy loss lead to an excessive fuel consumption, which, in turn, causes an excessive amount of deforestation in the nation as a result of societies cutting down bushes to meet the large demand for fuel woods. Numerous studies have shown that using biogas and the sun to cook and bake reduces indoor air pollution [8]. Sun cookers, however, are not as well accepted by society because they cannot be used to prepare meals indoors or at night. Furthermore, temperature variations might delay the baking process. Besides, until there is thermal storage for sustainable cooking techniques, preparing meals that need a lot of energy, such as injera, might be exceedingly challenging [9]. Nonetheless, a remarkable endeavor to implement injera baking biogas burners has been made possible by the Ethiopian National Biogas program. However, even if biogas is used for lighting, it has been demonstrated that using biogas for injera baking is challenging as the baking pan's ability to distribute heat from the hob is not usually well-designed or optimized. Additionally, there is a problem with support for the biogas period, which results in a shortage of fuel for the baking process [26]. Likewise, electric stoves are mostly found in urban areas, and it is difficult for those societies living far from the electric grid to bake their injera. Despite the efforts of the government of Ethiopia and other concerned organizations to distribute these improved cook stoves to the society, there is still a research gap in identifying other alternative injera baking stoves utilizing alternative energy sources.

Therefore, in rural areas, affordable, practical, and clean cooking and baking technologies are needed to reduce indoor air pollution and associated fuel consumption costs.

Among the different types of improved cook stoves, advanced biomass stoves are the subject of current research interest since they are more efficient, have lower emissions, are safer, and have high durability. The most recent advanced cook stoves are gasifiers and rocket stoves. These stoves reduce emissions by 40–75%, increase fuel consumption efficiency by 30%, and reduce the possibility of global warming by up to 40–60% [27]. However, these highly efficient cook stoves have not been designed or tested in such a way that whether they are suitable to cook stoves for baking injera is unknown in Ethiopia since injera baking is a highly energy-intensive process and requires special design techniques.

A biomass conversion device called a gasifier produces combustible gases from stable fuels, wood, manure, and agricultural waste. There are two combustion degrees in the gasifier range. Combustible gases are released during the first step of gas pyrolysis, which uses restricted air. In the second stage, sufficient air is mixed, and combustible gases burn on the pinnacle side of the range, providing thermal energy for the pan on top of the stove. The resultant petrol is a mixture of the incombustible gases nitrogen (N_2_) and carbon dioxide (CO_2_) with the combustible gases' hydrogen (H_2_), carbon monoxide (CO), and methane (CH_4_) [28]. Reviews have shown a few appealing qualities of gasifier stoves. During the combustion process, there is a uniform and constant flame, spotless flame management, a smoke-free and smooth combustion process, high performance, and a feasible interest-unfastened operation that lasts for the entire cooking time. As a result, there is a real need to create a biomass conversion method that is both enticing and environmentally friendly to replace the conventional wooden stoves that are still in use in rural communities for baking and cooking in developing countries like Ethiopia.

Ethiopia is a country with a rich cultural heritage and a long-standing tradition of using biomass as a source of energy for cooking. Injera, a staple food in Ethiopia, is traditionally baked on three-stone fires using wood or charcoal as fuel. However, this traditional method of cooking has several negative impacts on the environment, health, and economy of the country [29]. The use of wood and charcoal for cooking contributes significantly to deforestation, leading to soil erosion and loss of biodiversity. Moreover, the burning of these fuels releases harmful pollutants such as carbon monoxide, particulate matter, and black carbon, which not only contribute to air pollution but also have adverse health effects on the individuals using them. Furthermore, the reliance on wood and charcoal for cooking also has economic implications, as it is expensive and time-consuming to collect and purchase these fuels for daily use [30]. To address these issues, there has been a growing interest in the design and development of cleaner and more efficient cooking stoves that use renewable biomass as a fuel source. However, most of the research in this area has focused on developing stoves for general cooking purposes, neglecting the specific needs of the Ethiopian population for injera baking. This has resulted in a significant research gap in the design and development of a biomass gasifier cooking stove specifically tailored for injera baking [2]. The use of biomass gasifier stoves has been proven to be a viable alternative to traditional cooking methods, as they are more efficient and cleaner and use renewable biomass fuels such as agricultural waste and crop residues. These stoves use a process called gasification, where the biomass is heated in a low-oxygen environment, producing a clean-burning gas that can be used for cooking. With the proper design and development, these stoves can be adapted to cater to the specific needs of injera baking, such as the ability to regulate temperature and maintain a consistent heat source for the perfect texture and taste of the injera [31, 32].

The development of a biomass gasifier cooking stove for injera baking has the potential to bring numerous benefits to Ethiopia. It can help reduce deforestation and mitigate the negative impacts of traditional cooking methods on the environment. It can also improve the health and well-being of individuals by reducing exposure to harmful pollutants. Additionally, this stove can provide economic benefits by reducing the cost and time spent on collecting and purchasing fuels for cooking. Moreover, the development and widespread adoption of this stove can also create job opportunities in the manufacturing and maintenance of these stoves, leading to economic growth and development in the country [33]. The significant research gap in the design and development of a biomass gasifier cooking stove for injera baking in Ethiopia needs to be addressed. The potential benefits of such a stove are numerous and can have a significant positive impact on the environment, health, and economy of the country [34]. It is crucial for researchers, policymakers, and stakeholders to prioritize and invest in developing a suitable biomass gasifier stove for injera baking, which can bring sustainable and long-term benefits to the people and the nation as a whole [2, 35, 36].

Thus, this study aimed to design, develop, and test the performance of injera baking gasifier stoves powered by biomass fuel, to evaluate the degree of indoor air pollution levels to reduce health-related risks in societies.

## 2. Materials and Methods

### 2.1. Materials

To create dough and bake injera, teff grains were purchased, ground, and fermented. Strong waste biomass feedstock, similar to the dirt that was seen, was gathered, and its particle length (0.5–2.4 mm) was examined.

The 1.6 mm thick sheet metal that was utilized to make the injera baking gasifier series was purchased from suppliers.

Cooking implements, such as mitad and pan, were purchased from local suppliers.

Thermocouple used to measure baking temperature, indoor air emission tester.

A flue gas analyzer (Model: RASI 300C EiUK, Country: UK, company: Eurotron instrument UK ltd) was used to measure emission, and a thermal imaging camera was used to determine the uniformity of the baking temperature on the pan/mitad; these data were obtained from Bahir Dar Energy Center laboratories. An electronic balance was used to measure food and fuel mass during the performance testing process.

### 2.2. Methods

#### 2.2.1. Experimental Setup and Descriptions

**Figure 4:**
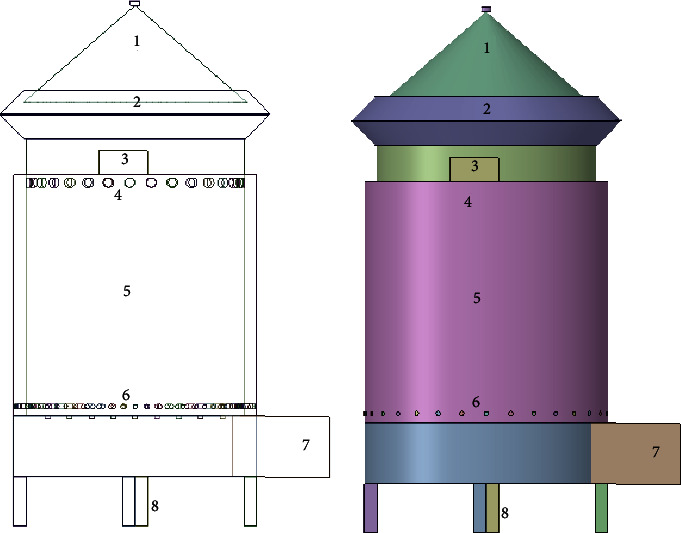
Injera baking gasifier stove model. (1) Lid of the pan, (2) pan, (3) fuel feed, (4) secondary air vent, (5) gasifier body, (6) primary air vent, (7) fan inlet, and (8) leg of the gasifier stove.

Figure 4 illustrates a schematic model of the injera baking gasifier stove. A gasifier stove was used for natural and forced convection. The stove has five major parts, namely, primary and secondary air, a gasifier reactor, a baking pan, fuel feed, and a charcoal outlet grate, as indicated in the 2D and 3D views.

The cooking pan was positioned underneath the pinnacle of the gasifier, where biomass gas was supplied and ignited. There is a shell and tube relationship in the gasifier reactor. Secondary air reached the gasifier's pinnacle through the shell and tube route, while primary air entered the stove's bottom. As pyrolysis proceeded, syngas was produced, reached the top of the gasifier, mixed with secondary air, and burned to heat the cooking pan. The injured baking pan was placed on the top of the gasifier stove above the secondary air inlet. After the baking process, the remaining pure chars were removed through the grate at the bottom of the gasifier stove. The injera was baked on the pan, which has a lid on the top that improves the texture of the injera.

#### 2.2.2. Preparation of Dough and Baking Process

White *teff* seeds were purchased from the Bahir Dar city market, cleaned with sieves, and then powdered using a mill. *teff* dough was prepared by mixing 60% water with 40% powdered *teff*, and this dough was fermented by adding yeast for 3-4 days to bake injera. After fermentation, the fermented dough was again mixed with hot water to dilute the mixture, after which the mixture was baked.

#### 2.2.3. Design of the Injera Baking Gasifier Stove

*(1) Energy Required*. Based on the energy needed to raise the dough's temperature to a desired level (practical warmth) and the amount of power needed to boil the water that evaporates during baking, the desired amount of heat strength to be supplied with the gasifier for injuring baking mushrooms was determined (latent heat) [10, 37, 38]. Thus, the total heat energy was determined using the following equation:

(1) $$\begin{array}{c} Q_{n}=Q_{\text{Sensible}}+Q_{\text{Latent}}, \end{array}$$

(2) $$\begin{array}{c} Q_{\text{Sensible}}=M_{\text{dough}}{\text{Cp}}_{\text{dough}}\left(T_{\text{boil}}-T_{\text{amb}}\right), \end{array}$$

(3) $$\begin{array}{c} Q_{\text{Latent}}=\left(M_{\text{dough}}-M_{\text{injera}}\right)h_{\text{fg}}. \end{array}$$

Therefore, the power *Q*_*np*_, the energy required to bake the injera, is given as follows:

(4) $$\begin{array}{c} Q_{\text{np}}=\frac{Q_{n}}{t}, \end{array}$$

where *Q*_*n*_ is the energy required to bake injera, *M*_dough_, mass of the dough (g), and *C*_*p*dough_, the specific heat of dough, which is assumed to be equivalent to the specific heat of water (4.2 kJ/kg°C);  *T*_boil_, boiling temperature (96°C); *T*_amb_, ambient temperature (20°C); *t*, average time for one injury to bake (2 min).

Generally, the average weight of one injera dough is approximately 450 g, 60% of the dough is water, and the remaining 40% of the dough is *Teflon* [10]. Thus, for this study, the average weight of one injury was 350 g.

In Ethiopia, the average family size is almost 5 persons per household. The societies of Ethiopia are well known for their hospitality to other people who are coming to their houses. They always celebrate with guests with different dishes and drinks. Therefore, the household consumes approximately 15–20 injera per day. The family-sized injeras are baked once in 3-4 day intervals (gap). Hence, considering these issues, the total family size of injeras is approximately 40–60, and the injuries are baked once every 3-4 days. Thus, the total energy demand is considered by taking a total injera of 40 in one baking process.

Fuel consumption, reactor diameter and height, cooking time, and airflow rate were determined based on Bilonio's work [39] as follows:

*(2) Fuel Intake Rate (FCR)*. The amount of electricity required in terms of fuel to be added to the cooker is represented by the FCR. This evolved into using equation (4).

(5) $$\begin{array}{c} \text{FCR}=\frac{\text{Qn}}{\text{Hvf}*\;\eta g}, \end{array}$$

where Qn is thermal energy required, Kcal/hr; HVf is fuel's heating value (15.59 MJ/kg); *η*_*g*_ is efficiency (21.8%)

*(3) Diameter of the Reactor*. This is a reference to the reactor's size, expressed in terms of the diameter of the cylinder's pass section where wood is burnt. It was decided to use equation (5).

(6) $$\begin{array}{c} {D=\left(\frac{1.27FCR}{\text{SGR}}\right)}^{0.5}, \end{array}$$

where *D* is the reactor's diameter in meters, FCR is the gas intake rate in kilograms per hour, and SGR is the specific gasification rate of biomass material, which ranges from 50 to 210 kg/m^2^-hr, taking into account a 40% protection element.

*(4) The Height of the Reactor*. The total distance from the top to the bottom of the reactor establishes how long the cooker would run on a single petrol loading. It is possible to calculate the reactor's height using the following equation:

(7) $$\begin{array}{c} H=\frac{\text{SGR}*T}{\rho }, \end{array}$$

where *H* is the reactor's length in meters, *m* is the SGR-specific gasification charge for wood, kg/m^2^/h, *T* is the time needed to consume wood, and *ρ* is the timber density, which is 54.6 kg/m^3^.

Air required for gasification: The amount of air required to gasify biomass. This is crucial in figuring out how big of a fan the reactor in gasifying wood has to be. Equation 8 is used to compute this. The proportion of wood biomass to air is within the range of 0.1 to 0.38, and the stoichiometry of air is also around 6.1 kg of air per kilogram of wood biomass [39]. A 0.3-equivalent ratio of wood (sawdust) gas was utilized to determine the air waft charge.

(8) $$\begin{array}{c} \text{AFR}=\frac{\varepsilon *\text{FCR}*\text{SA}}{\rho a}, \end{array}$$

where *ρ*a is the air density (1.25 kg/m^3^), FCR is the rate of intake of timber (kg/hr), SA is the stoichiometric air of timber (6.1 kg of air in step with kg of wood), and AFR is the air glide rate (m^3^/hr). *ε* is also the equivalency ratio of wooden, which is typically within the range of 0.1–0.38.

#### 2.2.4. Performance of the Injera Baking Stove

*(1) Boiling Water Look at This (WBT)*. The water boiling test is a crude representation of the cooking process that is meant to assist range designers in understanding how well power is transmitted from fuel to the cooking stove [40]. The product's mass in the cookware stove divided by its heat capacity, temperature exchange, evaporated water mass, and latent heat of evaporation about fuel mass and fuel energy is known as the proportion of thermal efficiency. Initially, each gas and pot that will be utilized in the test has been individually weighed. The pots were then in part filled with five liters of water and weighed once more. To this end, the preliminary temperature of the water was recorded before the range turned ignited to initiate the heating of the pot. Furthermore, the boiling temperature of the water was recorded.

When the gas becomes burned, the load of the water closing on the pot becomes recorded, and the thermal efficiency changed into determined the use of an equation.

(9) $$\begin{array}{c} \eta =\frac{\text{ Mn}*\text{Cp}*\left(\text{Tb}-\text{To}\right)+\text{Me}*L}{\text{Mf}*\text{Hv}}, \end{array}$$

where *η* denotes the thermal efficiency (%), Mn, the water mass in the pan (5 kg), and Cp, the water's specific heat (kj/kg/°C). In relation to the water's initial temperature (°C) and Tb, water temperature, at which it boils (°C), *L*, latent evaporation heat (kj/kg), Me, the mass of water evaporated (kg), *M*_*f*_ denotes the mass of fuel burned (2.8 kg), and Hv, fuel's heating value (15.59 Mj/kg).

*(2) Test for Controlled Cooking (CCT)*. The test between the cooking performance test and the basic water boiling test is the controlled cooking test [41]. The technique contrasts the amount of fuel used, the amount of leftover charcoal, and the amount of time needed to cook a meal on unique stoves. During this observation, it was seen that dust gas was being utilized for cooking in the area. It was chosen to use the locally available meal, injera, as the baking meal. Based on the VITA, every exam was chosen [12]. Technique for contrasting the modern era's fuel-saving efficiency with the traditional three-stone stove. For every sort of range, the check was conducted three times. Specific measurements were also made to determine the fuel wood's moisture content, the surrounding temperature, the amount of time needed to cook the dinner, the amount of time needed to light the fireplace, the first and final meal masses, the first and last gas timber loads, and the amount of leftover charcoal. The starting meal and fuel mass for this check were 5.6 kg and 2.8 kg, respectively.

#### 2.2.5. Heat Distribution of the Pan/Mitad

The heat distribution of the pan is very important for baking injera, which results in good texture and quality. The consistent distribution of heat on the pan determined the gasifier stove's performance. The uniformity of the heat distribution of the pan in the gasifier stove was determined by a thermal infrared thermal imager camera (company: Testo, country: USA model: Testo 875i, measuring range −30 to 350°C, infrared resolution 160 × 120 pixels), which analyzed the temperature profile of the yard/pan. The thermal imager camera has an accuracy of ±2, and collecting repeated measurements allows for error correction during measurement.

#### 2.2.6. Emission Level

The health-related emissions, such as CO, CO_2_, and PM concentrations, were quantified using an IAP meter (model: IAP meter 5000 series, company: ARC, country: USA, CO range: 0–1000 ppm, PM range: 0–60,000 *µ*g/m^3^) and a flue gas analyzer (model: RASI 300C EiUK, country: UK, company: Eurotron instrument UK ltd). The flue gas analyzer has an accuracy of ±1 to ±2, and collecting more than three measurements allows for error correction during measurement.

#### 2.2.7. Uncertainty Analysis

Statistical Package for the Social Sciences (SPSS) software was used to evaluate the statistical significance of each parameter using paired *t*-tests. The findings are shown as the mean with a *P* value (*P* < 0.05). Using IBM SPSS, regression analysis was carried out. The model that best suited the experimental data was chosen to use the correlation coefficient.

In electronic equipment temperature measurement, the greatest uncertainty when the value is low is ±2% of the reading value. With infrared thermometers, the typical uncertainty is about ±2% of the average reading.

### 2.3. Design Specifications

Table 1 provides a summary of all the layout parameters for the injera baking gasifier range. The energy requirement for baking the injera was 60.8 kW, as shown in Table 1. Based only on the energy need and cooking time, the ideal length of the injera baking gasifier range diameter and peak were determined to be 0.4 m and 0.48 m, respectively.

3.22 m^3^/hr of air was required for forced draft gasification. More smoke, however, emerged as the air pressure rose, indicating a change in the gasification process and a move towards the combustion approach. The design size also revealed that the forced draft gasification method's surface air speed changed to 0.1 m/s.

Parikh et al. [42] indicated that the fuel/air equivalency ratio sharply falls as the surface airspeed rises. On the other hand, a higher surface airspeed also resulted in a larger fuel consumption rate and, thus, a higher gasification temperature. The speed of the fuel phase grows, and convective cooling affects the method's extinction effects if the surface air pace rises beyond the limit.

## 3. Results and Discussions

### 3.1. Performance of Gasifier Stove

#### 3.1.1. Water Boiling Test

**Figure 5:**
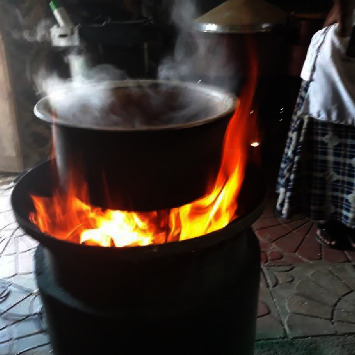
Water boiling test.

To determine how the new gasifier stove was constructed while modern baking techniques were being used, a controlled cooking check was established. When cooking at home, it is utilized to compare the overall performance of newer cookers to that of widely used stoves [29]. The water boiling test (WBT), as illustrated in Figure 5, was utilized in the current investigation to assess the gasifier stoves' overall performance with respect to the conventional WBT. Rather than measuring actual efficiency, the WBT primarily assesses heat transfer capability.

Throughout the high-energy stage (baking time), the hearth's thermal efficiency within the injera baking gasifier range was assessed. After lighting the gasifier stoves from the top to generate combustible gas, a blue flame appeared six minutes later. A total of 2.8 kg of sawdust biomass fuel was used in this overall performance test. The range burned nonstop for around 90 minutes.

The overall performance parameters of the gasifier range are shown in Table 2. The thermal performance of the injera baking gasifier range turned to 21.8%, similar to that pronounced with the aid of Guo et al. [43]. The charcoal content was 42%. One injury baking session lasted approximately 2-3 minutes, and the temperature ranged from 180 to 220°C.

#### 3.1.2. Controlled Cooking Test (CCT)

The prepared cook range's overall performance in controlled environments, as well as the utilization of locally accessible biomass fuels, meals, and pans, is evaluated using this efficacy test. Even though the cook dinner approach uses a regular meal on the stove, this test indicates how much gas is utilized. The stove's ability to save gas is evaluated by contrasting it with the three-stone range.  Table 3 presents an evaluation of the gas savings of the three-stone and injera baking gasifiers.  Table 3 illustrates that the injera baking gasifier stove had fuel and time savings efficiency of 86% and 38%, respectively, as compared to the three-stone stove baking system. Compared to the gasifier stoves, the three-stone stoves consumed more gas. Compared to the three-stone baking fuels (13 kg/hr), the precise petrol intake of the injera baking gasifier range (2.2 kg/hr) was found to be much lower. Also, it improved the previous studies [44].

**Figure 6:**
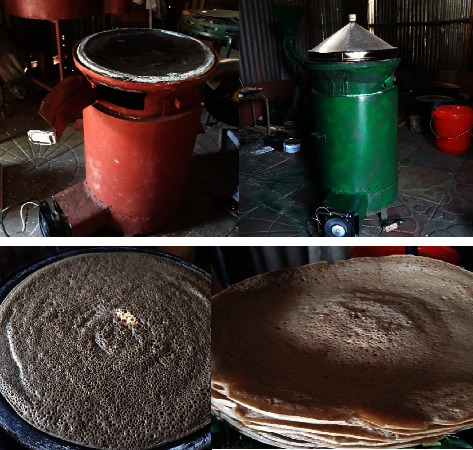
Prototype injera baking gasifier stove and texture of the produced injera.

The developed injera's fine: Figure 6 shows the injera's confirmed texture as well as the prototype of the injera baking gasifier burner. The quality of the produced injera was characterized by its visual appearance and smoothness. The injury texture and smoothness were very good and similar to those of conventional materials.

### 3.2. Heat Distribution Measurements

**Figure 7:**
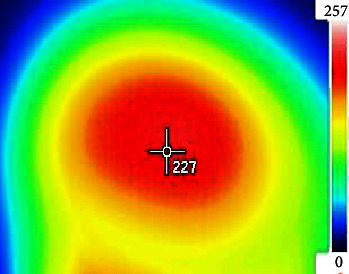
The mitad's heat distribution.

One of the most important factors influencing the temperature of mitad's surface is the pyrolysis temperature. The temperature within the mitad increased as the gasifier's temperature increased. The percentages of H_2_, CO, CO_2_, and CH_4_ in the generated gas are affected by temperature [30]. The influence of the airflow via the diameter of the secondary air drift vent and the gas flame is the heat distribution version. As shown in Figure 7, the temperature increases around the center of the pan as we move from the edge to the center. This is because the external air propelled the secondary air flow vent flame toward the center, even though the temperature exceeded the injera cooking range (180–220°C), which has a detrimental effect on the baking injera. As a result, any gaps that allow external air to enter the mad must be avoided. The temperature range for cooking injera in this novel design biomass gasifier stove was 180–220°C. This temperature range was optimized according to previous studies (650–800°C) [30]. This approach saves biomass and time.

### 3.3. Indoor Air Pollution

**Figure 8:**
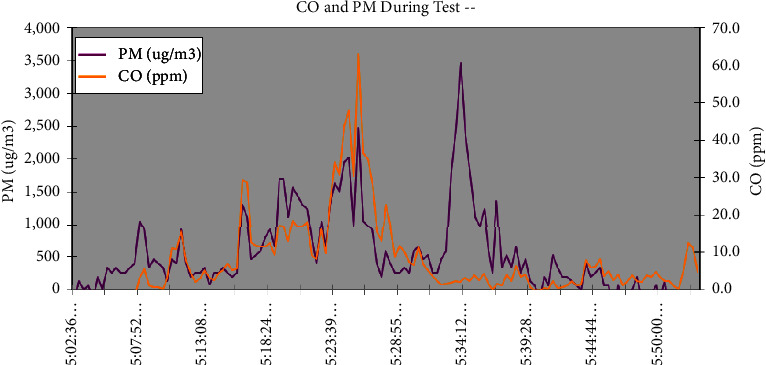
Concentrations of CO and PM with respect to baking time.

Reducing air pollution levels could potentially alleviate the impact of illnesses such as lung cancer, stroke, heart disease, asthma, and various chronic and acute respiratory disorders [45]. As shown in Figure 8, the concentrations of PM and CO decreased during the baking process, with average magnitudes of 608 *µ*g/m^3^ and 9.0 ppm, respectively. However, the concentrations of PM and CO increased during the middle of baking due to the increase in air availability, which led to incomplete combustion and the release of more CO.  Table 4 also reveals that the highest concentrations of PM and CO were 3,461 *µ*g/m^3^ and 63 ppm, respectively. The concentrations of PM and CO released by this gasifier stove are small compared to other previous studies [46, 47].

### 3.4. Baking Time

The amount of time required to bake the injera was employed as a benchmark to assess the range's overall effectiveness. In this newly designed biomass gasifier stove, it took 3 minutes on average to bake a single injera, whereas a three-stone fire took 4 minutes. Previous studies have shown that the injured baking time of a gasifier stove was 3.83 minutes [2]. As a result, the decrease obtained using the current design is more substantial than that obtained using alternative biomass-based stoves for the same application.

## 4. Conclusion

An injera baking gasifier stove on a laboratory scale was meticulously planned, constructed, and assessed. Sawdust and other stable waste biomass were converted to use as feedstock. Using recognized WBT and CCT procedures using observed dust as feedstock, the gasifier stove's performance was ascertained. The height and diameter of the designed injera baking gasifier stove had been 0.4 m and 0.48 m, respectively.

The power needed for one own family baking became 60.8 kW. The thermal performance of the injera baking gasifier range was 21.8%. Furthermore, the fuel-saving performance was changed to 86%, which was more than that of conventional stoves (mirt, gonze, lakech, and tikikil), which ranged from 25 to 50%. The baking temperature of the gasifier stoves turned to 196°C. The char yield of the gasifier range changed to 42%. 90 minutes was the burning period of 2.8 kg of biomass in the gasifier stoves, which is sufficient for Ethiopian households' whole cooking needs. Common values of CO and PM were 9 ppm and 608 *µ*g/m^3^, respectively. With this amended arrangement, the injera baking gasifier range's baking temperature ranges from 180 to 220°C and baking time was improved by 3 minutes.

The software model's projected results were not examined in this investigation. As a consequence, the outcomes that the finite element technique or CFD software predicts will be verified by both beginners and specialists in this finding.

For example, we may examine the stoichiometry of combustion air synthesis gas for several average air duct sizes for wood moisture content, gasification temperature, and airflow. It is also advised to research how thermal insulation affects temperature variations.

To strike a balance between cost-effectiveness, roasting quality, and efficiency, new gasifier designs are continuously being developed. Durability and domestic manufacture are significant additional variables.

## Figures and Tables

**Table 1:** Layout specifications.

Specifications	Unit	Value
Energy needed	KW	60.8
Fuel consumption rate	kg/hr	2.2
Reactor diameter	m	0.4
Height of the reactor	m	0.48
Burning time	hr	1.5
Gasification air flow rate	m^3^/hr	3.22
Superficial air velocity	m/s	0.1

**Table 2:** Using the WBT, the injera baking gasifier stove's thermal efficiency.

Parameter	Unit	Value
Fuel per batch	kg	3
Time to start up	min	5
Initial water mass	kg	5
Mass of water evaporated	kg	3.5
Time to boil water	min	10
Initial temperature	°C	20
Boiling temperature	°C	96
Burning time	min	90
Latent heat of evaporation	Kj/kg	2258
Specific heat of water	Kj/kg·°C	4.19
Maximum stove body temperature	°C	260
Maximum flame temperature	°C	750
Char yield	%	42
Thermal efficiency	%	21.8

**Table 3:** Control cooking test of three-stone and gasifier stoves.

1. CCT results: Stove 1	Units	Test 1	Test 2	Test 3	Mean	St. dev

Total weight of food cooked	g	22,300	22,200	22,200	22,233	58
Weight of char remaining	g	500	800	700	667	153
Equivalent dry wood consumed	g	404	265	193	288	107
Specific fuel consumption	g/kg	18	12	9	13	5
Total cooking time	min	85	90	115	97	16

2. CCT results: Stove 2	Units	Test 1	Test 2	Test 3	Mean	St Dev

Total weight of food cooked	g	22,300	22,200	22,200	22,233	58
Weight of char remaining	g	350	250	100	233	126
Equivalent dry wood consumed	g	52	42	28	41	12
Specific fuel consumption	g/kg	2	2	1	2	1
Total cooking time	min	50	60	70	60	10

Comparison of stove 1 and stove 2		% difference		*T* test	Sig @ 95%?	

Specific fuel consumption	g/kg	86%		3.98	YES	
Total cooking time	min	38%		3.35	YES	

**Table 4:** Average concentrations of PM and CO.

Concentration	Unit	Value
Average PM concentration	*µ*g/m^3^	608
Average CO concentration	ppm	9.0
Highest PM concentration	*µ*g/m^3^	3,461
Highest CO concentration	ppm	63.0
Highest 15-minute PM concentration	*µ*g/m^3^	1,067
Highest 15-minute CO concentration	ppm	20.1
Lowest 15-minute PM concentration	*µ*g/m^3^	159
Lowest 15-minute CO concentration	ppm	2.6

## Data Availability

The data that support the findings of this study can be obtained from the corresponding authors on request.

## References

[1] Benti N. E., Gurmesa G. S., Argaw T. (2021). The current status, challenges and prospects of using biomass energy in Ethiopia. *Biotechnology for Biofuels*.

[2] Adem K. D., Ambie D. A., Arnavat M. P., Henriksen U. B., Ahrenfeldt J., Thomsen T. P. (2019). First injera baking biomass gasifier stove to reduce indoor air pollution, and fuel use. *AIMS Energy*.

[3] World Health Organization (2010). *Preventing Disease through Healthy Environments. Exposure to Air Pollution: A Major Public Health Concern*.

[4] Lewis J. J., Pattanayak S. K. (2012). Who adopts improved fuels and cookstoves? A systematic review. *Environmental Health Perspectives*.

[5] Bhattacharya S. C., Leon M. A. *Prospects for Biomass Gasifiers for Cooking Applications in Asia*.

[6] Guta D. D. (2012). Assessment of biomass fuel resource potential and utilization in Ethiopia: sourcing strategies for renewable energies. *International Journal of Renewable Energy Resources*.

[7] Hassen A. A., Amibe D. A., Nydal O. J. Performance investigation of solar powered injera baking oven for indoor cooking.

[8] Liyew K. W., Habtu N. G., Louvet Y., Guta D. D., Jordan U. (2021). Technical design, costs, and greenhouse gas emissions of solar Injera baking stoves. *Renewable and Sustainable Energy Reviews*.

[9] Tesfay A. H., Kahsay M. B., Nydal O. J. (2014). Solar powered heat storage for Injera baking in Ethiopia. *Energy Procedia*.

[10] Hassen A. A., Kebede S. B., Wihib N. M. (2016). Design and manufacturing of thermal energy based injera baking glass Pan. *Energy Procedia*.

[11] Tesfay A. H., Kahsay M. B., Nydal O. J. (2014). Design and development of solar thermal Injera baking: steam based direct baking. *Energy Procedia*.

[12] Bruce N., Perez-Padilla R., Albalak R. (2000). Indoor air pollution in developing countries: a major environmental and public health challenge. *Bulletin of the World Health Organization*.

[13] Asfaw A. (2012). Sustainable household energy for addis ababa, Ethiopia. *Consilience*.

[14] Fullerton D. G., Bruce N., Gordon S. B. (2008). Indoor air pollution from biomass fuel smoke is a major health concern in the developing world. *Transactions of the Royal Society of Tropical Medicine and Hygiene*.

[15] Smith K. R., Samet J. M., Romieu I., Bruce N. (2000). Indoor air pollution in developing countries and acute lower respiratory infections in children. *Thorax*.

[16] Pope D. P., Mishra V., Thompson L. (2010). Risk of low birth weight and stillbirth associated with indoor air pollution from solid fuel use in developing countries. *Epidemiologic Reviews*.

[17] United Nations Environment Protection (2011). Integrated assessment of black carbon and tropospheric ozone. *Summary for Decision Makers, Environment*.

[18] Steubing B. (2011). Analysis of the availability of bioenergy and assessment of its optimal use from an environmental perspective.

[19] Dresen E., DeVries B., Herold M., Verchot L., Müller R. (2014). Fuelwood savings and carbon emission reductions by the use of improved cooking stoves in an afromontane forest, Ethiopia. *Land*.

[20] Beyene A. D., Koch S. F. (2013). Clean fuel-saving technology adoption in urban Ethiopia. *Energy Economics*.

[21] Heward-Mills D. (2004). *Table Of Contents Table Of Contents*.

[22] Geissler S., Hagauer D., Horst A., Krause M., Sutcliffe P. (2013). Biomass energy strategy: Ethiopia. *Energy Developments*.

[23] Yurnaidi Z., Kim S. (2018). Reducing biomass utilization in the Ethiopia energy system: a national modeling analysis. *Energies*.

[24] Lakew H., Hailu B., Hailu T., Carter S. (2017). *A Climate for Solar Power: Solutions for Ethiopia’s Energy Poverty*.

[25] IEA (2022). Ethiopian Energy Outlook. https://www.iea.org/articles/ethiopia-energy-outlook.

[26] Chandra A., Tiwari G. N., Srivastava V. K., Yadav Y. P. (1991). Performance evaluation of biogas burners. *Energy Conversion and Management*.

[27] Kshirsagar M. P., Kalamkar V. R. (2014). A comprehensive review on biomass cookstoves and a systematic approach for modern cookstove design. *Renewable and Sustainable Energy Reviews*.

[28] Iea Bioenergy (2015). *Task 33, Gasif. Biomass Waste*.

[29] Hailu A. T., Nega T., Hasan N., Worku E. (2023). Design and performance evaluation of low-emission injera baking biomass gasifier stove. *Biomass Conversion and Biorefinery*.

[30] Nega T., Habtu N. G., Tesfaye A., Melesse G. T., Aswossie E. (2022). Biomass energy conversion in a gasifier for injera baking mitad application. *Heliyon*.

[31] Tesfay A. H., Tsegay K., Kahsay M. B., Hailu M. H., Adaramola M. S. (2024). Performance comparison of three prototype biomass stoves with traditional and Mirt stoves for baking Injera. *Energy, Sustainability and Society*.

[32] Tariku A. (2020). Addis ababa science and technology university design, development and performance evaluation of an improved biogas injera baking stove design. *Development and Performance Evaluation of an Improved Biogas*.

[33] Abdella T. (2023). *Experimental Investigation of Biogas Injera Baking Stove Using Circular Ring Pipe Burner*.

[34] Okino J., Komakech A. J., Wanyama J., Ssegane H., Olomo E., Omara T. (2021). Performance characteristics of a cooking stove improved with sawdust as an insulation material. *Journal of Renewable Energy*.

[35] Adem K. D., Ambie D. A. (2017). A review of injera baking technologies in Ethiopia: challenges and gaps. *Energy for Sustainable Development*.

[36] Xiansheng G. (1993). Biomass domestic cooking gasifier stove for use in rural areas of developing countries. *Advances in Thermochemical Biomass Conversion*.

[37] Panwar N. L. (2009). Design and performance evaluation of energy efficient biomass gasifier based cookstove on multi fuels. *Mitigation and Adaptation Strategies for Global Change*.

[38] Lv P., Yuan Z., Ma L., Wu C., Chen Y., Zhu J. (2007). Hydrogen-rich gas production from biomass air and oxygen/steam gasification in a downdraft gasifier. *Renewable Energy*.

[39] Belonio A. T. (2005). *Rice Husk Gas Stove Handbook*.

[40] Bailis R., Ogle D., MacCarty N., Still D. (2007). *The Water Boiling Test (WBT) Version 3.0, Heal*.

[41] Berrueta V. M., Edwards R. D., Masera O. R. (2008). Energy performance of wood-burning cookstoves in Michoacan, Mexico. *Renewable Energy*.

[42] Parikh J., Channiwala S. A., Ghosal G. K. (2007). A correlation for calculating elemental composition from proximate analysis of biomass materials. *Fuel*.

[43] Guo F., Dong Y., Dong L., Guo C. (2014). Effect of design and operating parameters on the gasification process of biomass in a downdraft fixed bed: an experimental study. *International Journal of Hydrogen Energy*.

[44] Ahmad R., Energy T., Pemberton-pigott C., Abbas A. (2019). *Biomass Fuels*.

[45] WHO (2019). *Ambient (Outdoor) Air Quality and Health*.

[46] Ahmad R., Abbas A., Jufei W. (2021). Experimental and comparative study of Chinese commercial improved coal-fired cooking and space-heating stoves. *Environmental Science and Pollution Research*.

[47] Ahmad R., Zhou Y., Zhao N. (2019). Impacts of fuel feeding methods on the thermal and emission performance of modern coal burning stoves. *International Journal of Agricultural and Biological Engineering*.

